# Fields of a Bessel-Bessel light bullet of arbitrary order in an under-dense plasma

**DOI:** 10.1038/s41598-018-29694-y

**Published:** 2018-07-27

**Authors:** Yousef I. Salamin

**Affiliations:** 1 Max-Planck-Institut für Kernphysik, Saupfercheckweg 1, 69117 Heidelberg, Germany; 2 Department of Physics and Materials Science and Engineering Research Institute, American University of Sharjah, POB, 26666 Sharjah, United Arab Emirates

## Abstract

Considerable theoretical and experimental work has lately been focused on waves localized in time and space. In optics, waves of that nature are often referred to as light bullets. The most fascinating feature of light bullets is their propagation without appreciable distortion by diffraction or dispersion. Here, analytic expressions for the fields of an ultra-short, tightly-focused and arbitrary-order Bessel pulse are derived and discussed. Propagation in an under-dense plasma, responding linearly to the fields of the pulse, is assumed throughout. The derivation stems from wave equations satisfied by the vector and scalar potentials, themselves following from the appropriate Maxwell equations and linked by the Lorentz gauge. It is demonstrated that the fields represent well a pulse of axial extension, *L,* and waist radius at focus, *w*_0_, both of the order of the central wavelength *λ*_0_. As an example, to lowest approximation, the pulse of order *l* = 2 is shown to propagate undistorted for many centimeters, in vacuum as well as in the plasma. As such, the pulse behaves like a “light bullet” and is termed a “Bessel-Bessel bullet of arbitrary order”. The field expressions will help to better understand light bullets and open up avenues for their utility in potential applications.

## Introduction

Bessel beams were discovered more than three decades ago^1,2^ and have found numerous applications since, such as in optical trapping and tweezing^3–6^, precision drilling^7,8^, optical microscopy^9^ and laser acceleration^10^. Tightly-focused and temporally short pulses (or equivalently, ones that are of finite spatial extensions) are currently in great demand for many applications^11–16^. Central to the utility of such pulses is the need for analytic expressions for their electric and magnetic field components.

This paper aims to present analytic expressions for the fields of an ultra-short and tightly-focused Bessel pulse of arbitrary order, propagating in an under-dense plasma. The expressions, essentially describing a non-spreading wavepacket^17–21^, can be useful for many applications, including laser acceleration and high-harmonic generation (HHG) by colliding a tightly-focused and ultra-short pulse with a counter-propagating electron bunch^22^. In particular, a non-spreading wavepacket is highly desirable for laser-assisted atomic HHG^23–25^. Other plasma-based applications, such as the creation of plasma channels^26–29^ treated theoretically by particle-in-cell (PIC) simulations, may find the analytic expressions quite useful.

This work introduces orbital angular momentum into the description of a bessel-Bessel bullet^30,31^, for the first time. Among other things, opening up the Hilbert space of orbital angular momentum to be used to encode information in the fields of the bullets will boost efforts to utilize them in information transfer^32^.

Laser Bessel beam technology, based upon the use of axicon lenses, or combinations of annular slits and Fourier transforming lenses^2,32^ to achieve the required polarizations, is quite established now. However, experimental realization of the specific orbital angular momentum states of a Bessel-Bessel bullet may be challenging. Attempts to produce spatio-temporally localized Bessel-Bessel bullets experimentally may be guided by, and can benefit from, the recent work of Wise *et al*.^33^ on Bessel-Airy light bullets.

The basic assumption made in this work is that the response of the plasma to the fields of the laser pulse may be considered linear^34^. This can reliably be the case for non-relativistic pulse peak intensities (roughly, as long as *I* ≪ 10^18^ W/cm^2^, for a laser wavelength of 1 *μ*m). Under these conditions, Maxwell’s equations are entirely equivalent to^30,35–37^

1 $$({{\rm{\nabla }}}^{2}-\frac{1}{{c}^{2}}\frac{{{\rm{\partial }}}^{2}}{{\rm{\partial }}{t}^{2}}-{k}_{p}^{2}){\bf{A}}=0,$$

for the vector potential ***A***, together with a similar equation holding for the scalar potential, Φ, provided the two potentials are linked by the Lorentz gauge condition. Equation (1) has an effective plasma wavenumber *k*_*p*_ = *ω*_*p*_/*c*, in which *c* is the speed of light in vacuum and the plasma frequency is ${\omega }_{p}=\sqrt{{n}_{0}{e}^{2}/m{\varepsilon }_{0}}$, where *n*_0_ is the number density of the ambient electrons, *ε*_0_ is the permittivity of free-space and *m* and −*e* are the mass and charge, respectively, of the electron.

## Methods

### Change of coordinates

Equation (1) will be solved for the vector potential and the Lorentz condition will be used to obtain the associated scalar potential^30,31,37–42^. This process finally culminates in finding expressions for the ***E*** and ***B*** fields from the space- and time-derivatives of the potentials^35^. First, the Laplacian is expressed in cylindrical coordinates (*r*, *θ*, *z*). Next, assuming propagation along the *z*-axis, the following pair of new coordinates will be introduced in terms of the *z*- coordinate and the time

2 $$\eta =\frac{z+ct}{2};\,\,{\rm{a}}{\rm{n}}{\rm{d}}\,\,\zeta =z-ct.$$

For the centroid of the pulse (assumed to have been created at the origin of coordinates at *t* = 0 and to travel along the *z*- axis at approximately the speed of light) *z* ~ *ct* and, hence, *η* ~ *ct* and *ζ* ~ 0. In other words, *η* gives the position, on the propagation axis, of the centroid of the pulse at any time *t*, relative to the origin and *ζ* determines its coordinate relative to the moving centroid itself^37^. Employing the new variables, Eq. (1) transforms into

3 $$(\frac{1}{r}\frac{{\rm{\partial }}}{{\rm{\partial }}r}r\frac{{\rm{\partial }}}{{\rm{\partial }}r}+\frac{1}{{r}^{2}}\frac{{{\rm{\partial }}}^{2}}{{\rm{\partial }}{\theta }^{2}}+2\frac{{{\rm{\partial }}}^{2}}{{\rm{\partial }}\eta {\rm{\partial }}\zeta }-{k}_{p}^{2}){\boldsymbol{A}}=0.$$

The fields to be derived below possess radially- as well as azimuthally-polarized electric field components. Thus, the object to be described by these fields will be an ultra-short and tightly-focused laser pulse, which carries orbital angular momentum. In other words, it is the ultra-short, tightly-focused analogue of an arbitrary-order Bessel beam^1,2,32,42–45^.

### Truncated series solution

Letting $\hat{z}$ be a unit vector in the propagation direction and *k*_0_ = 2*π*/*λ*_0_ a central wavenumber corresponding to a central wavelength *λ*_0_, a one-component vector potential is put forth through the ansatz^42^

4 $${\boldsymbol{A}}(r,\theta ,\eta ,\zeta )=\hat{z}{a}_{0}a(r,\theta ,\eta ,\zeta ){e}^{i{k}_{0}\zeta },$$

with *a*_0_ a constant amplitude. The amplitude *a*(*r*, *θ*, *η*, *ζ*) is next synthesized from the Fourier components

5 $$a(r,\theta ,\eta ,\zeta )=\frac{1}{\sqrt{2\pi }}{\int }_{-\infty }^{\infty }{a}_{k}(r,\theta ,\eta ,k){e}^{ik\zeta }dk.$$

Using Eqs (4) and (5) in (3) yields an equation^30,31,37,40,41^

6 $$[\frac{1}{r}\frac{{\rm{\partial }}}{{\rm{\partial }}r}r\frac{{\rm{\partial }}}{{\rm{\partial }}r}+\frac{1}{{r}^{2}}\frac{{{\rm{\partial }}}^{2}}{{\rm{\partial }}{\theta }^{2}}+2i(k+{k}_{0})\frac{{\rm{\partial }}}{{\rm{\partial }}\eta }-{k}_{p}^{2}]\,{a}_{k}=0,$$

for each Fourier component *a*_*k*_. Solution to Eq. (6) will be sought using the standard textbook technique of separation of the variables. Thus, inserting *a*_*k*_(*r*, *θ*, *η*, *k*) = *f*_*k*_*F*(*r*)Θ(*θ*)*G*(*η*) into (6) and separating the variables, as usual, gives

7 $$\begin{array}{cc}\frac{{d}^{2}{\rm{\Theta }}}{d{\theta }^{2}}+{l}^{2}{\rm{\Theta }}=0;\,l=0,\pm \,1,\ldots ;\,\frac{dG}{d\eta }+\frac{i}{2}(\frac{{k}_{r}^{2}+{k}_{p}^{2}}{k+{k}_{0}})G=0; & {r}^{2}\frac{{d}^{2}F}{d{r}^{2}}+r\frac{dF}{dr}+({k}_{r}^{2}{r}^{2}-{l}^{2})F=0.\end{array}$$

Solutions to the above equations, which describe the physical situation of interest to us in this work, are: Θ ~ exp(*ilθ*) with *l* an integer, *G* a simple complex exponential and F ~ *J*_*l*_(*k*_*r*_*r*) an ordinary Bessel function of the first kind and order *l*. Furthermore, *k*_*r*_ is a separation constant, or radial index (not a radial wavenumber, because the wavevector does not have a radial component). In an experiment, the size of *k*_*r*_ will ultimately be determined by the size of the aperture used to produce the pulse, as will be described below.

The *k*^*th*^ Fourier component of the vector potential amplitude now takes on the form

8 $${a}_{k}\sim {f}_{k}{J}_{l}({k}_{r}r){e}^{il\theta }exp[\,-\,\frac{i}{2}(\frac{{k}_{r}^{2}+{k}_{p}^{2}}{k+{k}_{0}})\eta ],$$

with *f*_*k*_ independent of *η*, *θ* and *r*. For *f*_*k*_, we make the simple choice^46^

9 $${f}_{k}=\{\begin{array}{cc}\frac{\sqrt{2\pi }}{{\rm{\Delta }}k}, & \,|k|\le \frac{{\rm{\Delta }}k}{2};\,\\ 0, & \,{\rm{e}}{\rm{l}}{\rm{s}}{\rm{e}}{\rm{w}}{\rm{h}}{\rm{e}}{\rm{r}}{\rm{e}}.\end{array}$$

Implied in this choice is the assumption that the initial *wavepacket*, which evolves into the propagating pulse, consists of waves that possess a uniform spectrum, or distribution of wavenumbers^30,31,40^ of width Δ*k* and height $\sqrt{2\pi }/{\rm{\Delta }}k$. Note that this choice renders *a*_*k*_(0,0,0,*k*) = *f*_*k*_. Strictly speaking, this holds only for *l* = 0, in which case *f*_*k*_ has the Fourier transform *a*(0,0,0,*ζ*) = *f*(*ζ*) = sinc(*ζ*Δ*k*/2). The quantity | *f*(*ζ*)|^2^ represents the initial pulse intensity profile, with an approximate full-width-at-half-maximum ~2*π*/Δ*k*. Thus, it is plausible to adopt *L* = 2*π*/Δ*k* as representing the initial length (spatial extension) of the pulse in its propagation direction. On the other hand, the waist radius at focus, *w*_0_, will be shown shortly to be fixed by *x*_1,*l*_, the first zero of *J*_*l*_.

Putting (9) into (8) and the result back into (5) gives

10 $$a(r,\theta ,\eta ,\zeta )=\frac{{J}_{l}({k}_{r}r){e}^{il\theta }}{{\rm{\Delta }}k}{\int }_{-\frac{{\rm{\Delta }}k}{2}}^{\frac{{\rm{\Delta }}k}{2}}{\varphi }_{k}{e}^{ik\zeta }dk;\,\,{\varphi }_{k}(\eta )=\exp \,[\,-\,\frac{i}{2}(\frac{{k}_{r}^{2}+{k}_{p}^{2}}{k+{k}_{0}})\eta ].$$

Unfortunately, the integration in (10) cannot be carried out in closed analytic form. However, viewed as a function of *k*′ = *k* + *k*_0_, *ϕ*_*k*_ can be power-series expanded around *k*_0_, according to

11 $${\varphi }_{k}={\sum _{m=0}^{{\rm{\infty }}}\frac{{(k^{\prime} -{k}_{0})}^{m}}{m!}\frac{{{\rm{\partial }}}^{m}{\varphi }_{k}}{{\rm{\partial }}{k^{\prime} }^{m}}|}_{k^{\prime} ={k}_{0}}={\sum _{m=0}^{{\rm{\infty }}}\frac{{k}^{m}}{m!}{\varphi }_{0}^{(m)};{\varphi }_{0}^{(m)}(\eta )\equiv \frac{{{\rm{\partial }}}^{m}{\varphi }_{k}}{{\rm{\partial }}{k}^{m}}|}_{k=0}.$$

The integration in (10) may now be carried out in terms of incomplete gamma functions. Furthermore, on account of the fact that only the leading term(s) in (11) may contribute significantly in applications of interest, the series giving the full vector potential can be truncated to order *n* and written as

12 $${A}^{(n)}=\frac{{a}_{0}{J}_{l}({k}_{r}r){e}^{i({k}_{0}\zeta +l\theta )}}{2\pi /L}\sum _{m=0}^{n}\frac{{i}^{m+1}{\varphi }_{0}^{(m)}(\eta )}{m!{\zeta }^{m+1}}\,[{\rm{\Gamma }}\,(m+1,\frac{i\pi \zeta }{L})-{\rm{\Gamma }}\,(m+1,-\frac{i\pi \zeta }{L})],$$

in which Δ*k* has been replaced by 2*π*/*L*.

### Zeroth-order vector potential

From *A*^(*n*)^ follows a complete description for the fields of the ultra-short and tightly-focused pulse. The presence of *J*_*l*_ suggests that this object is the short-pulse analogue of an *l*^*th*^-order Bessel beam. The assumption will also be made that the first term in the series contributes the most, while terms beyond the first contribute negligibly^30^. Otherwise, one may still work with the truncated series to any desired order. The following expression for the zeroth-order vector potential follows from Eq. (12)

13 $${A}^{(0)}(r,\theta ,\eta ,\zeta )={a}_{0}{J}_{l}({k}_{r}r){j}_{0}(\frac{\pi \zeta }{L}){e}^{i{\phi }^{(0)}};\,\,{\phi }^{(0)}={\phi }_{0}+l\theta +{k}_{0}\zeta -\alpha \eta ,\,\,\alpha =\frac{{k}_{r}^{2}+{k}_{p}^{2}}{2{k}_{0}},$$

where the sinc function has been replaced by *j*_0_, the zero-order spherical Bessel function of the first kind, and *φ*_0_ is a constant phase. Note that in the case of a pulse containing a few cycles, *φ*_0_ plays the role of a carrier envelope phase (CEP).

**Figure 1:**
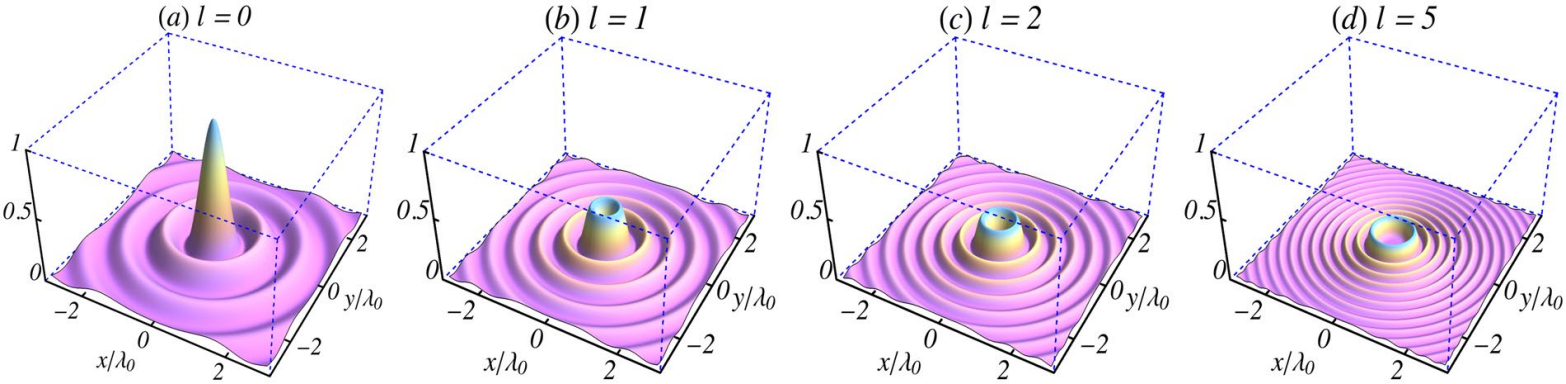
Surface plots of the initial (*t* = 0) vector potential *intensity profile* |*A*^(0)^/*a*_0_|^2^ in the focal plane (*z* = 0) of ultrashort (*L* = 1.5*λ*_0_) and tightly focused (*w*_0_ = 0.8*λ*_0_) laser pulses of central wavelength *λ*_0_ = 1 *μ*m, propagating in vacuum (*n*_0_ = 0). Other parameters used are: *φ*_0_ = 0 and *k*_*r*_ = *x*_1,*l*_/*w*_0_, where *x*_1,*l*_ is the first zero of *J*_*l*_(*x*).

**Figure 2:**
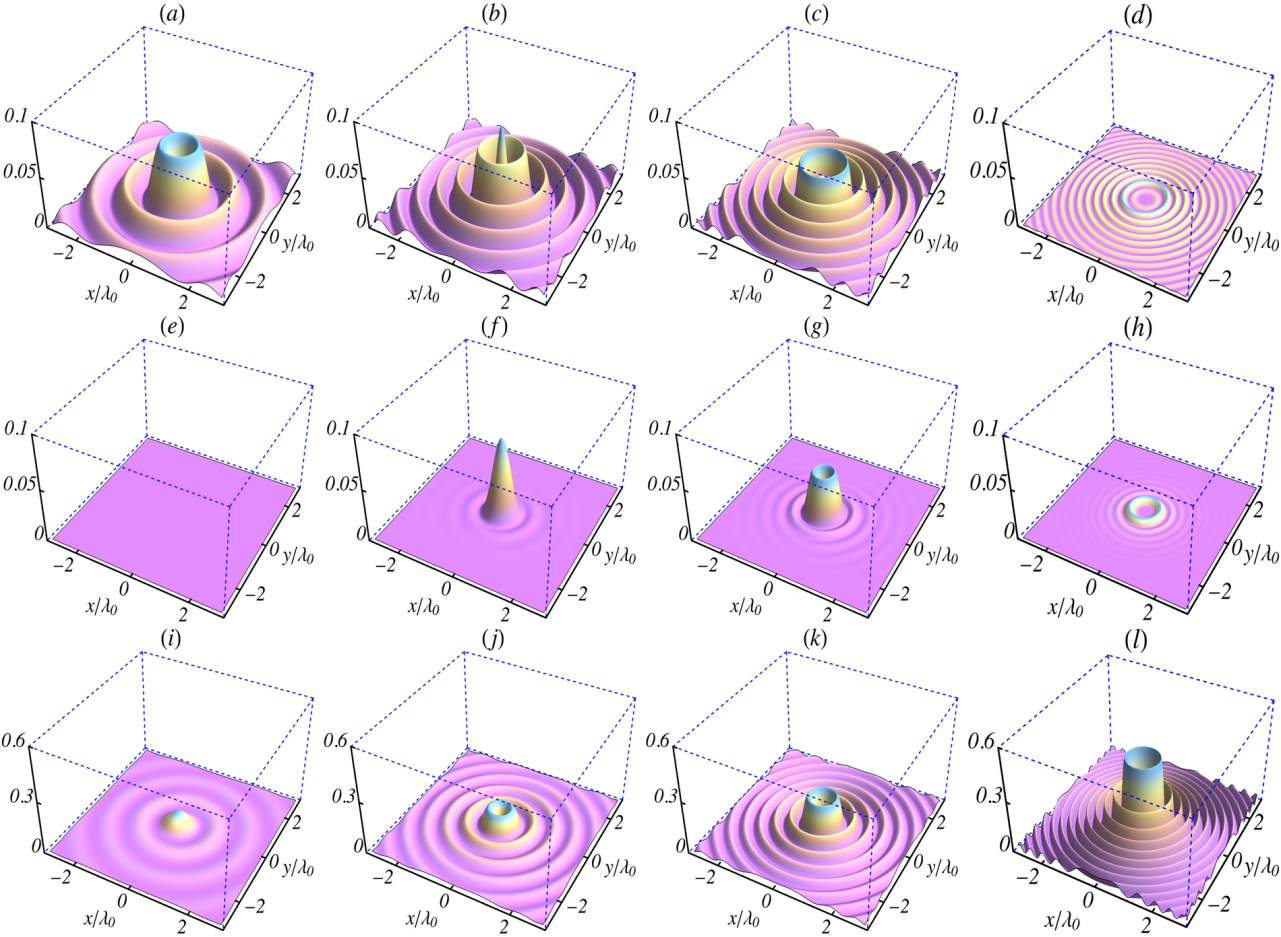
Surface plots of the initial (*t* = 0) intensity profiles in the focal plane (*z* = 0) of the electric field components given by Eqs (18 and 19). (**a**–**d**) ${|{E}_{r}^{\mathrm{(0)}}/{E}_{0}|}^{2}$, (**e**–**h**) ${|{E}_{\theta }^{\mathrm{(0)}}/{E}_{0}|}^{2}$ and (**i**–**l**) ${|{E}_{z}^{\mathrm{(0)}}/{E}_{0}|}^{2}$. The columns (left to right) are for *l* = 0, 1, 2 and 5, respectively. The parameters used are: *L* = 1.5*λ*_0_, *w*_0_ = 0.8*λ*_0_, *λ*_0_ = 1 *μ*m, *n*_0_ = 0, *φ*_0_ = 0 and *k*_*r*_ = *x*_1,*l*_/*w*_0_, where *x*_1,*l*_ is the first zero of *J*_*l*_(*x*).

**Figure 3:**
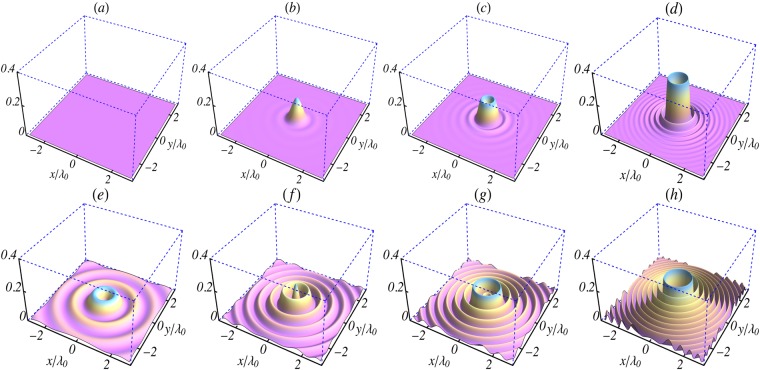
Surface plots of the initial (*t* = 0) intensity profiles in the focal plane (*z* = 0) of the magnetic field components given by Eqs (21 and 22). (**a**–**d**) ${|c{B}_{r}^{\mathrm{(0)}}/{E}_{0}|}^{2}$ and (**e**–**h**) ${|c{B}_{\theta }^{\mathrm{(0)}}/{E}_{0}|}^{2}$. The columns (left to right) are for *l* = 0,1,2 and 5, respectively. The parameters used are: *L* = 1.5*λ*_0_, *w*_0_ = 0.8*λ*_0_, *λ*_0_ = 1 *μ*m, *n*_0_ = 0, *φ*_0_ = 0 and *k*_*r*_ = *x*_1,*l*_/*w*_0_, where *x*_1,*l*_ is the first zero of *J*_*l*_(*x*).

**Figure 4:**
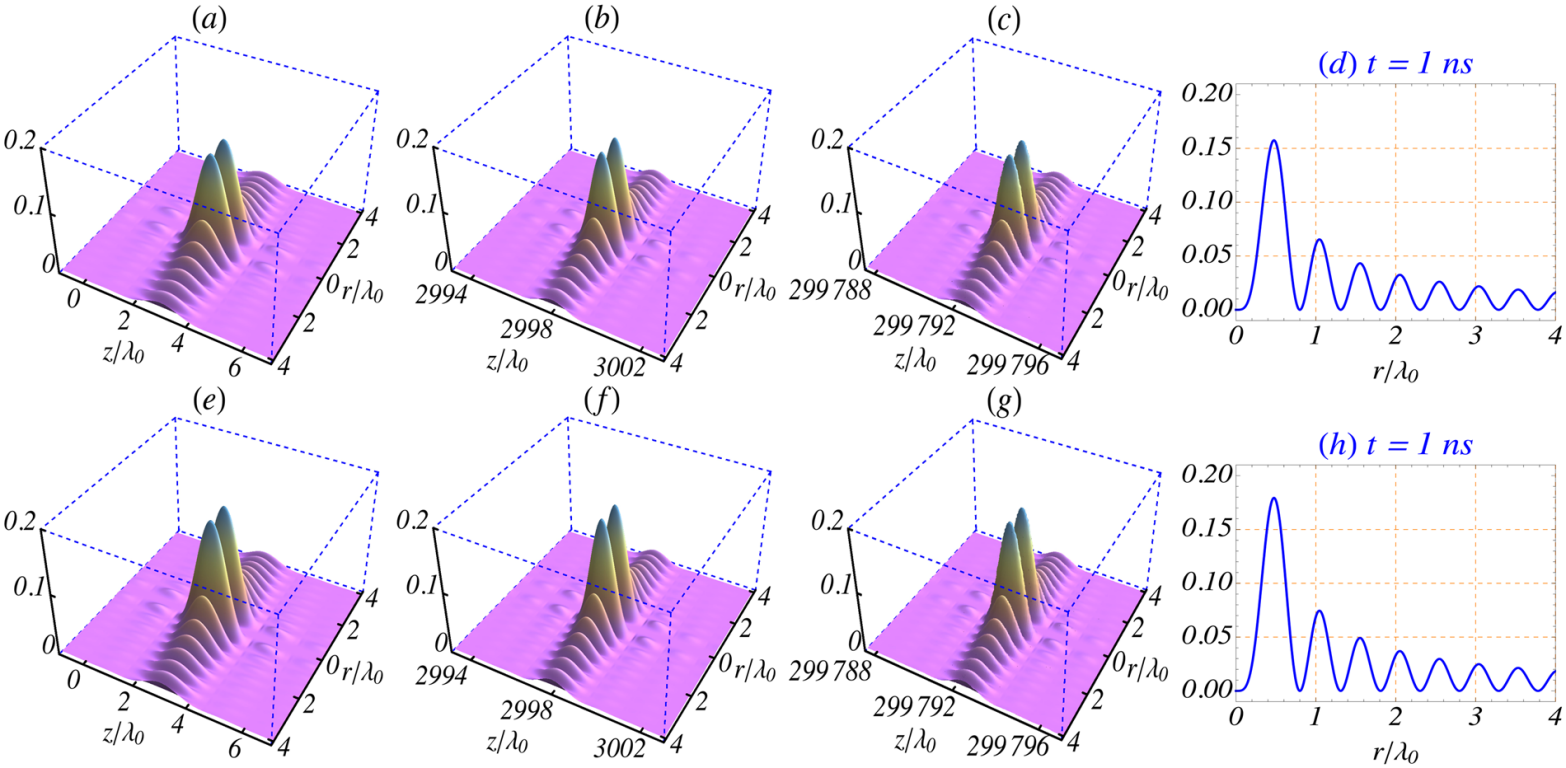
(**a**–**c**) Surface plots of the axial electric field intensity profile ${|{E}_{z}^{\mathrm{(0)}}/{E}_{0}|}^{2}$, in the *z* − *r* plane, for the ultra-short and tightly-focused analog of the order-2 Bessel beam (*l* = 2). These are snapshots taken at *t* = 10 fs, 10 ps and 1 ns, respectively, during propagation of the pulse in vacuum. (**e**–**g**) Show the same during propagation in an under-dense plasma, with ambient electron density *n*_0_ = 10^20^ cm^−3^. (**d**) and (**h**) show radial variation of the intensity profile at *t* = 1 ns at the plane defined by *z* = *ct*. The remaining parameters used are: *L* = 1.5*λ*_0_, *w*_0_ = 0.8*λ*_0_, *λ*_0_ = 1 *μ*m, *φ*_0_ = 0 and *k*_*r*_ = *x*_1,2_/*w*_0_, where *x*_1,2_ is the first zero of *J*_2_(*x*).

For a pulse created at *t* = 0, with its centroid at the origin of coordinates, ${|{A}^{\mathrm{(0)}}/{a}_{0}|}^{2}={J}_{l}^{2}({k}_{r}r)$. Surface plots of this quantity, in the focal plane, are shown in Fig. 1, for *l* = 0, 1, 2 and 5, exhibiting all the expected properties of the square of *J*_*l*_. It should also be borne in mind that the axial extension of the pulse, *L*, was determined above by the first zero of *j*_0_. The temporal width of the pulse may be taken as *τ* ~ L/c, while the waist radius at focus, *w*_0_, is such that *k*_*r*_*w*_0_ = *x*_1,*l*_, the first zero of *J*_*l*_. With the waist radius *predetermined* as *w*_0_ = 0.8*λ*_0_ in Figs 1–4, the radial index *k*_*r*_ → *k*_*r*,*l*_, i.e., it takes on different values for the different orbital angular momentum states indexed by *l*. In an experiment, the radial indices and waist radii may alternatively be determined from knowledge of the radius *r*_*a*_ of the aperture employed to produce and observe the intensity patterns. The radius *w*_*n*_ containing the *n*^*th*^ observed ring would be such that *k*_*r*,*l*_*w*_*n*_ = *x*_*n*,*l*_, where *x*_*n*,*l*_ is the *n*^*th*^ zero of *J*_*l*_. Using *w*_*n*_ ~ *r*_*a*_, one gets *k*_*r*,*l*_ ~ *x*_*n,l*_/*r*_*a*_. Had Fig. 1, in which *r*_*a*_ = 3*λ*_0_, been produced experimentally, this procedure would have resulted in the values listed in the third column of Table 1 for the patterns displayed, respectively, in Fig. 1(a–d). These radial index values lead to the waist radii at focus listed in the fourth column of the same Table.

**Table 1:** Radial index values *k*_*r*,*l*_ ~ *x*_*n*,*l*_/*r*_*a*_ and waist radii *w*_0,l_ ~ (*x*_1,*l*_/*x*_*n*_,_*l*_)*r*_*a*_ that would be associated with intensity patterns similar to those of Fig. 1, produced using an aperture of radius *r*_*a*_ = 3*λ*_0_, which would admit *n* rings in a typical case.

*l*	*n*	*k*_*r*,*l*_ ~ *x*_*n*,*l*_/*r*_*a*_$[{{\boldsymbol{\lambda }}}_{{\bf{0}}}^{{\boldsymbol{-}}1}]$	*w*_0,l_ ~ (*x*_1,*l*_/*x*_*n*_,_*l*_)*r*_*a*_ [*λ*_0_]
0	3	2.88	0.834
1	4	4.44	0.863
2	5	5.99	0.858
5	8	10.6	0.827

Note that *w*_0,*l*_ > *w*_0_ = 0.8*λ*_0_, for all cases considered.

It will be demonstrated below that both *L* and *w*_0_ stay roughly fixed in magnitude during propagation of the pulse, thus making the pulse essentially diffraction-free and dispersion-free. This may all be traced back to the absence of nonlinear terms in Eq. (1). The above features qualify the pulse for being a “light bullet”^33,46–52^.

## Results

### The fields

The electric and magnetic fields of the pulse, explicit knowledge of which is required for many analytic and numerical calculations, follow from ***E*** = −∇Φ − ∂***A***/∂*t* and ***B*** = ∇ × ***A***, most appropriately in cylindrical coordinates. The electric field has three components: radial *E*_*r*_, azimuthal *E*_*θ*_ and axial *E*_*z*_. These may, respectively, be found from^31,37^

14 $${E}_{r}=-\,\frac{{c}^{2}}{s}\frac{{\rm{\partial }}}{{\rm{\partial }}r}(\frac{{\rm{\partial }}A}{{\rm{\partial }}z})-\frac{{c}^{2}}{{s}^{2}}(\frac{{\rm{\partial }}A}{{\rm{\partial }}z})\frac{{\rm{\partial }}}{{\rm{\partial }}r}(\frac{1}{a}\frac{{\rm{\partial }}a}{{\rm{\partial }}t}),$$

15 $${E}_{\theta }=-\,\frac{{c}^{2}}{s}\frac{{\rm{\partial }}}{r{\rm{\partial }}\theta }(\frac{{\rm{\partial }}A}{{\rm{\partial }}z})-\frac{{c}^{2}}{{s}^{2}}(\frac{{\rm{\partial }}A}{{\rm{\partial }}z})\frac{{\rm{\partial }}}{r{\rm{\partial }}\theta }(\frac{1}{a}\frac{{\rm{\partial }}a}{{\rm{\partial }}t}),$$

16 $${E}_{z}=-\,\frac{{\rm{\partial }}A}{{\rm{\partial }}t}-\frac{{c}^{2}}{s}\frac{{{\rm{\partial }}}^{2}A}{{\rm{\partial }}{z}^{2}}-\frac{{c}^{2}}{{s}^{2}}(\frac{{\rm{\partial }}A}{{\rm{\partial }}z})\frac{{\rm{\partial }}}{{\rm{\partial }}z}(\frac{1}{a}\frac{{\rm{\partial }}a}{{\rm{\partial }}t}),$$

where *s* = *ick*_0_ − (1/*a*)∂*a*/∂*t*. Moreover, the magnetic field has two components: radial, *B*_*r*_ and azimuthal, *B*_*θ*_, which follow, respectively, from

17 $${B}_{r}=\frac{1}{r}\frac{{\rm{\partial }}A}{{\rm{\partial }}\theta },\,\,{\rm{a}}{\rm{n}}{\rm{d}}\,\,{B}_{\theta }=-\,\frac{{\rm{\partial }}A}{{\rm{\partial }}r}.$$

According to Eqs (14–16) the electric field components, after some tedious algebra and with arguments of all the Bessel functions temporarily suppressed, are

18 $${E}_{r}^{(0)}=-\,{E}_{0}(\frac{{k}_{r}}{{k}_{0}})\frac{{e}^{i{\phi }^{(0)}}}{R}[{Q}_{1}\,{j}_{0}(\frac{\pi \zeta }{L})+\frac{\cos (\pi \zeta /L)}{\zeta }]\,[\frac{{J}_{l-1}-{J}_{l+1}}{2}],$$

19 $${E}_{\theta }^{(0)}=-\,il{E}_{0}(\frac{{k}_{r}}{{k}_{0}})\frac{{e}^{i{\phi }^{(0)}}}{R}\,[{Q}_{1}\,{j}_{0}(\frac{\pi \zeta }{L})+\frac{\cos (\pi \zeta /L)}{\zeta }]\frac{{J}_{l}}{{k}_{r}r},$$

20 $${E}_{z}^{(0)}=\frac{{E}_{0}}{{k}_{0}}{e}^{i{\phi }^{(0)}}\{[{Q}_{2}+\frac{{Q}_{3}}{R}-\frac{{Q}_{1}{Q}_{4}}{{R}^{2}}]\,{j}_{0}(\frac{\pi \zeta }{L})+\frac{1}{\zeta }[1-\frac{2{Q}_{1}}{R}-\frac{{Q}_{4}}{{R}^{2}}]\,\cos (\frac{\pi \zeta }{L})\}{J}_{l},$$

Furthermore, Eq. (17) give the following expressions for the associated magnetic field components

21 $${B}_{r}^{(0)}=il{E}_{0}(\frac{{k}_{r}}{c{k}_{0}}){e}^{i{\phi }^{(0)}}{j}_{0}(\frac{\pi \zeta }{L})\frac{{J}_{l}}{{k}_{r}r},$$

22 $${B}_{\theta }^{(0)}=-\,{E}_{0}(\frac{{k}_{r}}{c{k}_{0}}){e}^{i{\phi }^{(0)}}{j}_{0}(\frac{\pi \zeta }{L})\,[\frac{{J}_{l-1}-{J}_{l+1}}{2}].$$

In Eqs (18)–(22) *E*_0_ = *ck*_0_*a*_0_, and

23 $${Q}_{1}=i{k}_{0}-\frac{1}{\zeta }-\frac{i\alpha }{2};\,\,\,\,{Q}_{2}=i{k}_{0}-\frac{1}{\zeta }+\frac{i\alpha }{2};\,\,\,\,R={Q}_{2}+\frac{\pi }{L}cot(\frac{\pi \zeta }{L}),$$

24 $${Q}_{3}=\frac{2{Q}_{1}}{\zeta }+{k}_{0}^{2}+\frac{{\pi }^{2}}{{L}^{2}}+\frac{\alpha (\alpha -4{k}_{0})}{4};\,\,\,\,{Q}_{4}=-\,\frac{1}{{\zeta }^{2}}+\frac{{\pi }^{2}}{{L}^{2}}{csc}^{2}(\frac{\pi \zeta }{L}).$$

Equations (18–24) give the field components of a pulse propagating in vacuum by taking the limit *k*_*p*_ → 0. They also have the expected limits in the case of a zero-order Bessel pulse^36^, for which the components ${E}_{\theta }^{\mathrm{(0)}}$ and ${B}_{r}^{\mathrm{(0)}}$ are absent, while ${E}_{r}^{\mathrm{(0)}}$ and ${B}_{\theta }^{\mathrm{(0)}}$ vanish identically at all points on the *z*- axis. These components have the *hollow* intensity profiles displayed in Figs 2 and 3. Note also that for the *l* = 1 pulse only the intensity profile corresponding to ${E}_{z}^{\mathrm{(0)}}$ is hollow. On the other hand, all profiles are hollow for *l* ≥ 2, due to the vanishing of *J*_*l*_ at *r* = 0, for all *l* ≥ 1.

### Propagation characteristics

Using the second of Eq. (13) one gets a general expression for the wavevector of the pulse in cylindrical coordinates, namely

25 $${\bf{k}}={\rm{\nabla }}{\phi }^{(0)}={k}_{\theta }\hat{\theta }+{k}_{z}\hat{{\bf{z}}};\,\,{k}_{\theta }=\frac{l}{r},\,\,{k}_{z}={k}_{0}-\frac{\alpha }{2}.$$

According to Eq. (25) the wavevector does not have a radial component and wavefronts (surfaces of constant phase) are helices of fixed radii *r*. In this sense the bullet carries orbital angular momentum, with the index *l* labeling different states in the relevant Hilbert space^42^. In general, an effective frequency for the bullet may be obtained from *ω* = −∂*φ*^(0)^/∂*t* = *c*(*k*_0_ + *α*/2), from which the dispersion relation ${(\omega /c)}^{2}-{k}_{z}^{2}=2{k}_{0}\alpha$, follows immediately. From this, in turn, one gets an effective wavenumber $k=\omega /c=\sqrt{{k}_{z}^{2}+{k}_{r}^{2}+{k}_{p}^{2}}$. For the fields to describe a propagating pulse, the axial wavevector must be positive definite, *k*_*z*_ > 0. According to the last of Eqs. (25) this condition is equivalent to the requirement *k*_*r*_ < 2*k*_0_ = 4*π*/*λ*_0_. All cases in Table 1 satisfy this condition and, hence, should describe pulses propagating through an aperture of radius *r*_*a*_ = 3*λ*_0_.

Key propagation characteristics of the pulse are illustrated in Fig. 4. The figure displays surface plots, in the *z*-*r* plane, of the intensity profile |*E*_*z*_/*E*_0_|^2^ for an ultra-short and tightly-focused Bessel pulse of order *l* = 2, at the instants (following generation at *t* = 0) of *t* = 10 fs, 10 ps and 1 ns. (a–c) display behavior during propagation in vacuum and (e–g) in an under-dense plasma of ambient electron density *n*_0_ = 10^20^ cm^−3^. From both sets of figures, one sees that the pulse propagates without distortion, neither by dispersion nor by diffraction. Over the time interval from 0 to 1 ns, the centroid of the pulse covers a distance, in the propagation direction, of about 30 cm, with the pulse-shape remaining almost intact. (d) and (h) show variations with the radial distance *r* of the axial intensity profile in the plane *z* = *ct*. Comparison of (d) with (h) reveals that the presence of a plasma background alters the central intensity maximum by roughly 10%, compared to its vacuum-based counterpart, for the parameter set used. For this particular set of parameters, the fields are enhanced by the presence of the plasma.

## Discussion

Fields of an ultra-short and tightly-focused Bessel pulse of arbitrary order have been derived. The expressions presented and discussed in this paper are fully analytic, but approximate. They have been arrived at strictly analytically from a one-component vector potential polarized along the propagation direction of the pulse, together with a scalar potential linked to the said vector potential by the Lorentz gauge. Vector potential of the pulse of finite extension has been synthesized, like a wavepacket, from Fourier components of a uniform wavenumber distribution. The full vector potential has been given as a power-series expansion and the leading (zeroth-order) term only has been used to derive the electric and magnetic field components reported in this paper. Intensity profiles of the field components have been discussed and shown to propagate like those of a laser bullet, without distortion by dispersion or diffraction. Because of the presence of two Bessel functions in the expression giving the zeroth-order vector potential, the pulse deserved the designation as a “Bessel-Bessel laser bullet”.

As the figures presented above show, fields of the orbital-angular-momentum-carrying Bessel-Bessel bullets have complicated intensity distributions, with some of them possessing sizable background radial oscillations, in addition to the main/central peaks. To rid a typical pulse of its background oscillations and make it useful for such applications as laser-acceleration, considerable pre- and post-pulse work may be required, which can alter the intensity and impact the efficacy of the process in question. Furthermore, in applications like HHG, the polarization and carrier envelope phase of the pulse play important roles. Control of the polarization-related effects and CEP stability may turn out to be challenging for the laser technology that would aim for experimental realization and ultimate utilization of a Bessel-Bessel bullet.

## References

[1] Durnin J (1987). Exact solutions for nondiffracting beams. I. The scalar theory. JOSA B.

[2] Durnin J, Miceli JJ, Eberly JH (2010). Diffraction-free dielectric particles by cylindrical vector beams. Opt. Express.

[3] Kozawa Y, Sato S (2010). Optical trapping of micrometer-sized dielectric particles by cylindrical vector beams. Opt. Express.

[4] Tian B, Pu J (2011). Tight focusing of a double-ring-shaped, azimuthally polarized beam. Opt. Lett..

[5] Kotlyar VV, Kovalev AA, Porfirev AP (2016). An optical tweezer in asymmetrical vortex Bessel-Gaussian beams. J. Appl. Phys..

[6] Friese MEJ, Nieminen TA, Heckenberg NR, Rubinsztein-Dunlop H (1998). Optical alignment and spinning of laser-trapped microscopic particles. Nature.

[7] Duocastella M, Arnold CB (2012). Bessel and annular beams for materials processing. Laser Photon. Rev..

[8] Malinauskas M (2016). Ultrafast laser processing of materials: from science to industry. Light: Science & Applications.

[9] Comin, A. *et al*. *CLEO: 2015, OSA Technical Digest* (online), paper SW1H.5 (Optical Society of America, 2015).

[10] Salamin YI (2017). Electron acceleration in vacuum by a linearly-polarized ultra-short tightly-focused THz pulse. Phys. Lett. A.

[11] C*entral Laser Facility:* http://www.clf.stfc.ac.uk/CLF/12248.aspx.

[12] E*xtreme Light Infrastructure:* http://www.eli-beams.eu/.

[13] Ursescu D (2016). Laser beam delivery at ELI-NP. Rom. Rep. Phys..

[14] Turcu ICE (2016). High-field physics and QED experiments at ELI-NP. Rom. Rep. Phys..

[15] Ciappina MF (2017). Attosecond physics at the nanoscale. Rep. Prog. Phys..

[16] Jirka M, Klimo O, Vranic M, Weber S, Korn G (2017). QED cascade with 10 PW-class lasers. Sci. Rep..

[17] Malomed BA, Mihalache D, Wise F, Torner L (2005). Spatiotemporal optical solitons. J. Opt. B.

[18] Mihalache D (2012). Linear and nonlinear light bullets: recent theoretical and experimental studies. Rom. J. Phys..

[19] Malomed BA (2016). Multidimensional solitons: Well-established results and novel findings. Eur. Phys. J. Special Topics.

[20] Malomed B, Torner L, Wise F, Mihalache D (2016). On multidimensional solitons and their legacy in contemporary Atomic, Molecular and Optical physics. J. Phys. B: At. Mol. Opt. Phys..

[21] Mihalache D (2017). Multidimensional localized structures in optical and matter-wave media: A topical survey of recent literature. Rom. Rep. Phys..

[22] Li J-X, Chen YY, Hatsagortsyan KZ, Keitel CH (2017). Angle-resolved stochastic photon emission in the quantum radiation-dominated regime. Sci. Rep..

[23] Protopapas M, Keitel CH, Knight PL (1997). Atomic physics with super-high intensity lasers. Rep. Prog. Phys..

[24] Agostini P, DiMauro LF (2004). The physics of attosecond light pulses. Rep. Prog. Phys..

[25] Kohler MC, Pfeifer T, Hatsagortsyan KZ, Keitel CH (2012). Harmonic generation from laser-driven vacuum. Adv. At. Mol. Phys..

[26] Willingale L (2006). Collimated Multi-MeV Ion Beams from High-Intensity Laser Interactions with Underdense Plasma. Phys. Rev. Lett..

[27] Willingale L (2009). Relativistic Transparent Regime through Measurements of Energetic Proton Beams. Phys. Rev. Lett..

[28] Fan J, Parra E, Milchberg HM (2000). Resonant self-trapping and absorption of intense Bessel beams. Phys. Rev. Lett..

[29] Meng, W., Salamin, Y. I. & Keitel, C. H. Electron acceleration by a radially-polarized laser pulse in a plasma micro-channel. (submitted). 10.1364/OE.27.000557 30696140

[30] Salamin YI (2017). Fields of an ultrashort tightly focused radially polarized laser pulse in a linear response plasma. Phys. Plasmas.

[31] Salamin YI (2015). Fields and propagation characteristics in vacuum of an ultrashort tightly focused radially polarized laser pulse. Phys. Rev. A.

[32] Dudley A, Lavery M, Padgett M, Forbes A (2013). Unraveling Bessel Beams. Opt. Photon. News.

[33] Chong A, Renninger WH, Christodoulides DN, Wise FW (2010). Airy–Bessel wave packets as versatile linear light bullets. Nature Photonics.

[34] Sprangle P, Esarey E, Krall J, Joyce G (1992). Phys. Propagation and guiding of intense laser pulses in plasma. Phys. Rev. Lett..

[35] Jackson, J. D. *Classical Electrodynamics*, 3rd edition (Wiley, 1998).

[36] Salamin YI (2017). Approximate fields of an ultra-short, tightly-focused, radially-polarized laser pulse in an under-dense plasma: a Bessel-Bessel light bullet. Opt. Express.

[37] Esarey E, Sprangle P, Pilloff P, Krall J (1995). Theory and group velocity of ultrashort, tightly focused laser pulses. JOSA B.

[38] McDonald, K. T. http://puhep1.princeton.edu/kirkmcd/examples/axicon.pdf.

[39] Li J-X, Salamin YI, Hatsagortsyan KZ, Keitel CH (2016). Fields of an ultrashort tightly-focused laser pulse. JOSA B.

[40] Salamin YI (2015). Simple analytical derivation of the fields of an ultrashort tightly focused linearly polarized laser pulse. Phys. Rev. A.

[41] Salamin YI, Li J-X (2017). Electromagnetic fields of an ultra-short tightly-focused radially-polarized laser pulse. Opt. Commun..

[42] McDonald, K. T. http://puhep1.princeton.edu/˜kirkmcd/examples/bessel.pdf.

[43] Milione G (2015). Using the nonseparability of vector beams to encode information for optical communication. Opt. Lett..

[44] Fu S, Zhang S, Gao C (2016). Bessel beams with spatial oscillating polarization. Sci. Rep..

[45] Davis LW (1979). Theory of electromagnetic beams. Phys. Rev. A.

[46] Wang, R. *Introduction to Orthogonal Transforms: With Applications in Data Processing and Analysis* (Cambridge University, 2012).

[47] Di Trapani P (2003). Spontaneously Generated X-Shaped Light Bullets. Phys. Rev. Lett..

[48] Siviloglou GA, Broky J, Dogariu A, Christodoulides DN (2007). Observation of Accelerating Airy Beams. Phys. Rev. Lett..

[49] Zhong W-P, Belić M, Huang T (2010). Three-dimensional Bessel light bullets in self-focusing Kerr media. Phys. Rev. A.

[50] Urrutia JM, Stenzel RL (2016). Helicon waves in uniform plasmas. IV. Bessel beams, Gendrin beams and helicons. Phys. Plasmas.

[51] Mendoza-Hernández J, Arroyo-Carrasco M, Iturbe-Castillo M, Chávez-Cerda S (2015). Laguerre–Gauss beams versus Bessel beams showdown: peer comparison. Opt. Lett..

[52] Volke-Sepulveda K, Garcés-Chávez V, Chávez-Cerda S, Arlt J, Dholakia K (2002). Orbital angular momentum of a high-order Bessel light beam. J. Opt. B: Quantum Semiclass. Opt..

